# Enhanced Vibration Isolation with Prestressed Resonant Auxetic Metamaterial

**DOI:** 10.3390/ma14226743

**Published:** 2021-11-09

**Authors:** Adrien Pyskir, Manuel Collet, Zoran Dimitrijevic, Claude-Henri Lamarque

**Affiliations:** 1 LTDS UMR-CNRS 5513, École Centrale de Lyon, 69134 Écully, France; manuel.collet@ec-lyon.fr; 2 Stellantis, 78140 Vélizy-Villacoublay, France; zoran.dimitrijevic@stellantis.com; 3 LTDS UMR-CNRS 5513, École Nationale des Travaux Publics de l’État, 69120 Vaulx-en-Velin, France; Claude-Henri.LAMARQUE@entpe.fr

**Keywords:** metamaterial, vibration isolation, resonance, auxetic, bandgap

## Abstract

Metamaterials designate structures with properties exceeding bulk materials. Since the end of the 1990s, they have attracted ever-growing attention in many research fields such as electromagnetics, acoustics, and elastodynamics. This paper presents a numerical and experimental study on a locally resonant auxetic metamaterial for vibration isolation. The designed materials combine different mechanisms—such as buckling, local resonances, and auxetism—to generate enhanced isolation properties. This type of structure could help to improve the isolation for machines, transportation, and buildings. First, the static properties of the reference and resonant structures are compared. Dispersion curves are then analysed to describe their periodic dynamic behaviour. An experimental validation carried out on a specially designed test bench is then presented and compared to corresponding finite structure simulation. As a result, huge bandgaps are found for the resonant case and strong isolation properties are also confirmed by the experimental data.

## 1. Introduction

Researchers’ interest for metamaterials has been regurarly increasing for more than two decades now, with uplifting applications like invisibility cloaks [1] or perfect lens [2]. Their multidisciplinary aspect contributes to the spreading effort to improve these frequently periodic structures, exhibiting properties unobserved in conventional materials [3].

Even though other types of systems can be used and combined to display exotic properties, the majority of concepts encountered in literature harness resonance phenomena to achieve these properties, whether it is in electromagnetism [4], acoustics [5], or elastodynamics [6]. More recently, other fields like plasmonics [7] and biosensors [8] have also seen interesting resonance-based metamaterials enlarge their range of solutions. One of the main features of resonance is that it can cancel low frequency waves and thus embodies a compact alternative to conventional vibration isolation materials [9]. However, its effective range is often narrow and requires substantial mass addition to be applied at low frequencies [10].

Auxetic metamaterials are another class of frequently studied structures, characterised by a negative Poisson’s ratio. As such, they expand in one or several transverse directions under axial traction, and, conversely, tend to contract transversally when axially compressed. Such architectures have been known for quite a long time [11,12], but at first only their static properties—like effective Poisson’s ratio and stiffness modulus—were studied. Their dynamic characteristics began to gain attraction at the end of the nineties [13]. Just like numerous metamaterials, they can feature frequency bands where no waves can propagate, called bandgaps. For obvious reasons, bandgaps are extremely interesting for vibration isolation and to find bandgaps as wide and low frequency possible with a compact design is a paramount ambition in this study.

Some auxetic metamaterials also harness buckling to exhibit unique static and dynamic properties, with promising applications in vibration isolation [14,15].

Other papers tried to combine such structures with resonant inclusions to increase the isolation performance of the system [16,17]. Such mechanism is sometimes labelled as hyperdamping [18].

This paper focuses on the performance of such structures for vibration isolation and studies an enhanced patented design combining buckling and local resonances. In particular, our structures’ effective frequency range can be tuned by increasing their compression state, and substantial weight can be withstood without impairing their performance. As a result, this could lead to applications in vibration isolation where a compact, low-frequency, and wideband solution is required, such as vibrating machines in industry, silent blocks, and seismic insulation.

The first part presents the studied geometries then describes the numerical methods used. The following part deals with the infinite plate assumption, with static compression computations and dispersion analysis of both geometries. It sets the reference results for the finite structure study, detailed in the last part. Numerical and experimental stress–strain compression curves are first compared, after which a frequency domain analysis is performed to compare both samples and check the coherence between experimental and numerical data.

## 2. Materials and Methods

### 2.1. Design of Periodic Pattern

**Figure 1:**
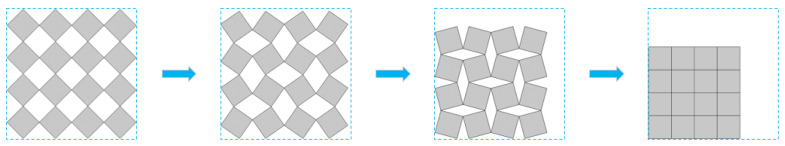
Base mechanism for rotating square structures. Configurations for different angles between adjacent squares (Respectively from left to right: 45°, 30°, 15°, 0°).

The geometry taken as reference in this study is derived from the rotating squares design, a quite classical structure used by numerous authors for more than two decades [19]. The auxetic mechanism governing its deformation is a simple geometric one: each 2D polygon can rotate with respect to its neighbours. All these rotations change the global size of the structure in both directions (See Figure 1).

**Figure 2:**
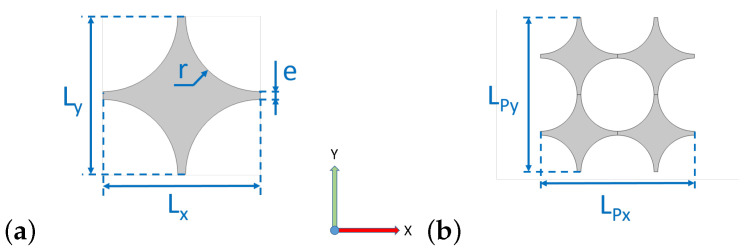
Reference structure $S_{0}$: (**a**) unit cell and (**b**) periodic pattern.

However, such a design requires point contacts between polygons, which is hard to implement, practically. Some articles present systems really close to it [20], but many others [14,16,17] rather opt for a simple modification. Indeed, replacing the square holes between polygons (see the first configuration in Figure 1) with round holes fixes the point contact problem and the associated stress concentration near sharp angles. The modified geometry is shown in Figure 2.

**Figure 3:**
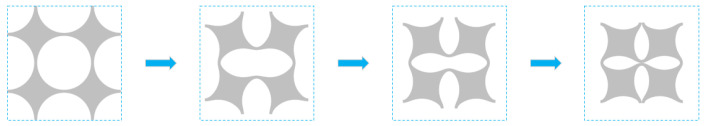
Mechanical behaviour during compression of periodic pattern $S_{0}$. Configurations for different values of deformation (Respectively from left to right: 0%, 10%, 20%, ≈30%).

Although this geometry exhibits the same auxetic properties as the original one, their respective mechanical behaviour strongly differs. Instead of linear rotations until complete closing as described by Figure 1, the polygons’ rotation now triggers the intermediate thin ligaments’ buckling, which is intrinsically non-linear (See Figure 3).

Simplifying this array, it can be seen as an array of masses (the square parts) linked to their neighbours by springs (the ligaments). Analytical models describing the dynamic behaviour of such arrays have been known for a long time [21]. Another model, attributed to Milton and Willis [22], shows that adding locally resonant masses opens a subwavelength bandgap in the dispersion diagram [23,24]. Based on this result and the many exemples of locally resonant metamaterials in the literature [5], a new design with resonant inclusions, referred to here as $S_{R}$, has been proposed and patented. Although a similar design was presented by Cui and Harne [17], it is the first time it is applied to elastodynamics and harness phemomena such as buckling and resonance.

**Figure 4:**
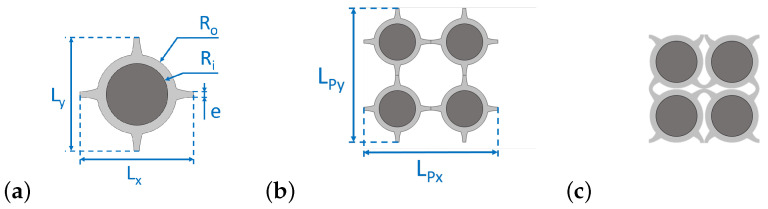
Resonant design $S_{R}$ with its characteristic dimensions for $R_{i}=3$ mm: (**a**) unit cell, (**b**) periodic pattern, and (**c**) pattern at ≈30% compression level.

Practically, the design in Figure 4 is achieved adding a disk centred on the square shape centre and a second concentric disk inside the first one. The first disk is designed to optimise the unused space when the material is compressed (see the last step of Figure 3), while the second acts as the local resonator.

For the sake of comparison, the same initial geometrical parameters will be defined as follows for both the initial configuration $S_{0}$ and the resonant one $S_{R}$: $L=L_{x}\left(\varepsilon =0\right)=L_{y}\left(\varepsilon =0\right)=11.2$ mm, e=1.2 mm. The radius *r* (see Figure 2a) is directly derived from previous parameters through $r=\frac{L-e}{2}=5$ mm. Moreover, all the results in this study are obtained for an out-of-plane thickness $h_{z}=40$ mm which corresponds to the experimental samples’ thickness. Additional parameters for $S_{R}$ sample are the inner radius $R_{i}=3$ mm and the outer radius $R_{o}=4.2$ mm, so as to keep the same thickness *e*. It should also be mentioned that the cells dimensions $L_{x}\left(\varepsilon \right)$ and $L_{y}\left(\varepsilon \right)$ vary with the structure deformation $\varepsilon =\frac{L_{y}\left(0\right)-L_{y}\left(\varepsilon \right)}{L_{Py}\left(\varepsilon \right)}$, as they are not equal to each other anymore—though very close—when ε>0.

Therefore, the unit cells shown here (Figure 2a and Figure 4a) are not periodic anymore after buckling. Four cells should be considered to obtain the actual periodic pattern. The characteristic lengths of this pattern along *X* and *Y* are respectively $L_{Px}\left(\varepsilon \right)=2L_{x}\left(\varepsilon \right)$ and $L_{Py}\left(\varepsilon \right)=2L_{y}\left(\varepsilon \right)$, as explained by Figure 2b and Figure 4b.

### 2.2. Design of Finite Structures

**Figure 5:**
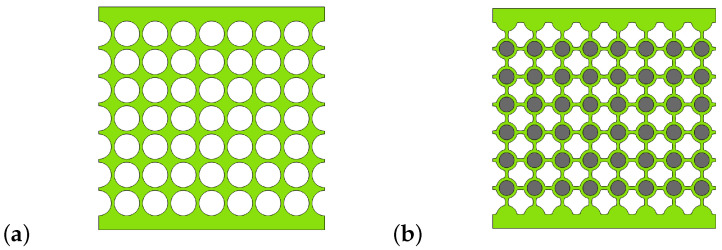
Finite structures designed from 8 × 7 (**a**) $S_{0}$ cells and (**B**) $S_{R}$ cells.

For the dispersion curves to be accurate and close to the behaviour of a finite structure, one would want the latter to have as many cells as possible. For the sake of practicality and feasibility, we wanted the samples’ size to be about 100×100 mm^2^ in the XY plane. From the unit cell’s characteristic sizes defined in previous section, we chose to make samples with 8×7 unit cells, as shown in Figure 5. Their length along *Z* axis is chosen to be 40 mm, in order to avoid out-of-plane deformations.

**Figure 6:**
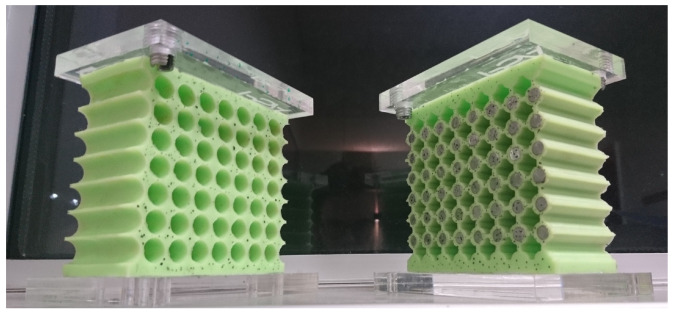
Samples $S_{0}$ (**left**) and $S_{R}$ (**right**) used in this study.

In order to have viable samples, several details had to be considered. First, in order to prevent the silicone from breaking due to stress concentration, the top and bottom boundaries run through the centre of the unit cells (instead of through the ligaments like the left and right boundaries). Secondly, to ensure the clamped–clamped boundary condition on the top and bottom sides, a 5 mm bulk layer of material is added on these boundaries. Finally, rigid plates made out of PMMA are then fixed on these layers in order to prevent any transverse displacements as well as disbonding with the bench plates and to facilitate the mounting on the bench (the PMMA plate can be screwed to the bench plates). The final samples are shown in Figure 6.

These samples have been made by molding a two-phase silicone rubber (Zhermack Elite Double 32, Badia Polesine, Italy) at room temperature. The $S_{0}$ mold was in aluminium, with steel rods embedded in it to make the circular holes in the material. As for the $S_{R}$, its mold was 3D printed out of ABS material, with a set of cylinders and a set of "plus" shaped rods to make both the inclusions’ locations and the voids, respectively, in the final design.

### 2.3. Materials

The matrix material chosen for the entire study is a silicone elastomer, exhibiting a very large yield strength. For the sake of simplicity, a linear elastic material model is used for the computations. For the resonator, the material should be very stiff and very dense compared to the silicone, as property contrast is the key point to enhance the resonators’ effect. Steel has thus been chosen, for it is cheap, easily processed and its properties, detailed in Table 1, fit well these requirements.

**Figure 7:**
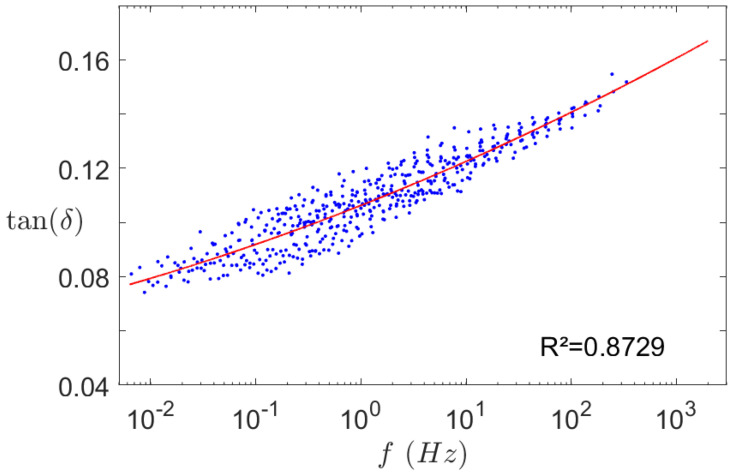
Damping coefficient master curve of the silicone at 22 °C. • Experimental data and 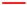 quadratic interpolation curve.

A Dynamic Mechanical Analysis (DMA) was also performed to evaluate the dynamic damping coefficient of the silicone. This test is used to characterise the mechanical behaviour of a material as a function of temperature and excitation frequency. It is particularly useful for polymers, which are very sensitive to thermal or frequency variations. Even though this test has little interest for the static study, it is essential for the dynamic one, as it gives the mechanical behaviour of the material over a wide frequency range, thanks to the time-temperature superposition. The frequency range in this study is only a few thousands hertz but it can be great to check if some properties like the damping coefficient vary along this range. Figure 7 shows this variation for a temperature of 22 °C. It can be seen that the damping coefficient, close to 10%, does not vary much in the study frequency range. Therefore, a constant coefficient η=10% is input in the dynamic model; results are showed in Section 3.2.2.

### 2.4. Experimental Setup

**Figure 8:**
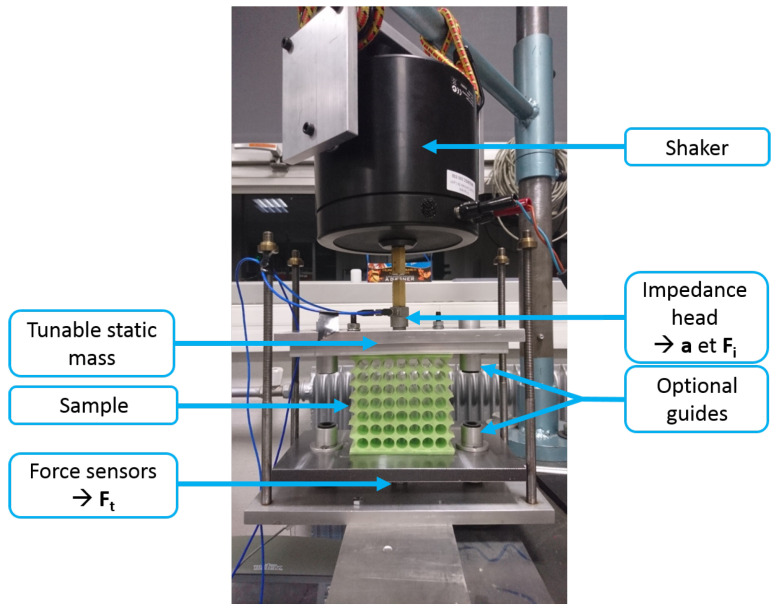
Test bench for vibration isolation measurements.

A test bench was specially designed to observe the isolation properties of the metamaterials. As can be seen in Figure 8, the sample is rigidly fixed between two plates. The bottom plate is bound to the bed table via three ICP^®^ force sensors 208C02 from PCB Piezotronics. On the top plate, masses can be added to achieve different states of strain. In our case, the minimum total mass m=600 g (static mass and top plate) is used for the measurements. A shaker Brüel & Kjær type 4809 is suspended above the top plate and bound to it through a aluminium rod and an ICP^®^ impedance head 288D01 from PCB Piezotronics. This sensor measures both the input force and acceleration.

As indicated in Figure 8, guides can be added to ensure the unidirectionality of the displacements. However, guides tend to transmit vibrations, sometimes greater than the vibrations transmitted through the sample, so the results presented here were all obtained without guides. The drawback is that it can foster out-of-plane modes, unaccounted for in the computations. Nevertheless, for small deviations like the ones in this study, the samples are stiff enough for the excitation to be predominantly longitudinal.

### 2.5. Numerical Methods—Brillouin Zone

For periodic structures, the Brillouin Zone (BZ) is defined as the periodic pattern transposed in the reciprocal space, that is to say the wavenumber space [21]. Every pattern in the structure is identical, so that all the eigenmodes can be obtained from the BZ data only. Moreover, Brillouin showed that symmetries in the BZ can be used to further reduce the calculation domain and to find a minimal zone called Irreducible Brillouin Zone (IBZ).

**Figure 9:**
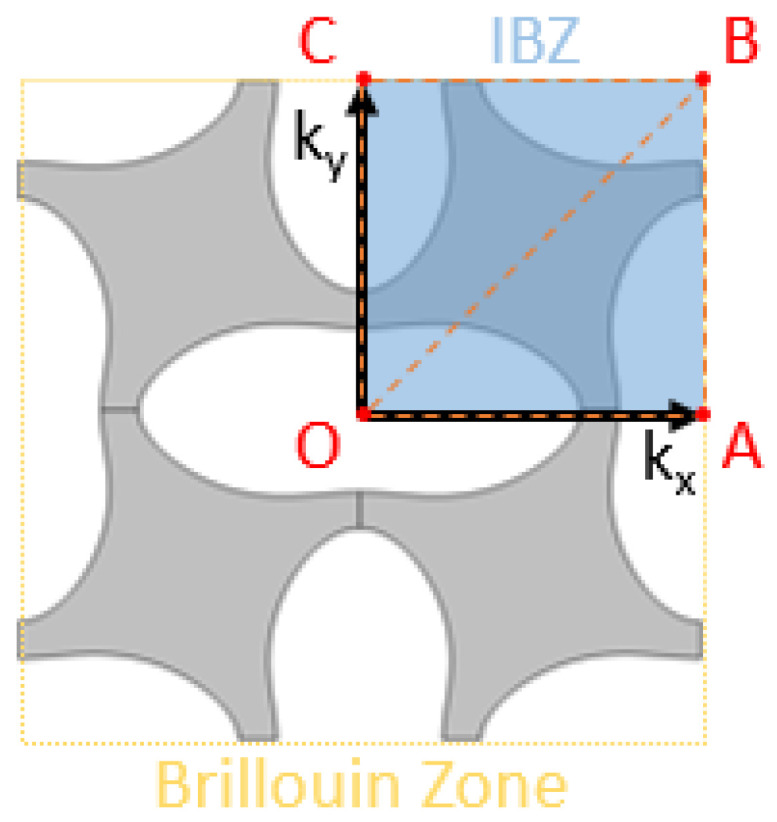
BZ and IBZ depiction for a 2D pattern.

In Figure 9, the IBZ is seen to amount to a quarter of the BZ area. In undeformed configuration, this IBZ could even be reduced by half (i.e., OAB area only), but as hinted before, as soon as ε>0, the symmetry along OB axis is not valid anymore.

In the end, the IBZ allows one to determine the dynamic behaviour of the whole structure with an optimised computational cost, as the waves in the IBZ and in the entire structure are considered to be the same. Further simplification is achieved by computing only the IBZ contour—that is to say OA, AB, BC, and OC—visible in Figure 9. The eigenmodes are then usually presented in graphs plotting the frequency versus the wavenumber, called band diagrams—or dispersion diagrams. This type of graph is particularly useful to highlight bandgaps and thus the isolating performance of the metamaterial.

Although most articles compute only the IBZ contour, it is interesting to mention that other papers found that depending on the shape of the IBZ, extrema can exist inside the domain [25,26]. Thus if preliminary detection of bandgaps can be achieved through the IBZ contour, validating the exact bandgaps frequency range will require a full sweep of the IBZ. This study implements five branches whose bounds are defined as follows:

 **OA:**  $k_{x}\in \frac{\Delta {}^{\circ}}{L_{x}}$ et $k_{y}=0$
 **AB:**  $k_{x}=\frac{\pi }{L_{x}}$ et $k_{y}\in \frac{\Delta {}^{\circ}}{L_{y}}$
 **BC:**  $k_{x}\in \frac{\Delta {}^{\circ}}{L_{x}}$ et $k_{y}=\frac{\pi }{L_{y}}$
 **OC:**  $k_{x}=0$ et $k_{y}\in \frac{\Delta {}^{\circ}}{L_{y}}$
 **OB:**  $k_{x}\in \frac{\Delta {}^{\circ}}{L_{x}}$ et $k_{y}=k_{x}\frac{L_{x}}{L_{y}}$

where Δ°=[0,π] is the sweep range.

### 2.6. Numerical Methods—Wave Finite Element Method

Wave Finite Element Method (or *WFEM*) is a classical method to compute the dispersion diagram of periodic structures. It is a hybrid method combining the analytical approach to finite elements in order to model complex structures with limited computational cost. An overview of the method is presented here, while a detailed description can be found in [27,28]. The method uses the Floquet–Bloch theorem which states that displacements on the boundaries of an L-periodic pattern (periodic pattern where L is the space period) are governed by the relation:

(1) $$u\left(\omega ,x\right)=\tilde{u}\left(\omega ,x\right)e^{jkx}$$

where $\tilde{u}$ is L-periodic. When applied to vibration propagation in a 2D pattern periodic along *X* and *Y*, whose size is $L_{x}\times L_{y}$, and for boundaries only, the equation gives:

(2) $$\left\{\begin{array}{c} u_{R}=u_{L}e^{jk_{x}L_{x}}\\ u_{T}=u_{B}e^{jk_{y}L_{y}} \end{array}\right.$$

where $u_{•}={\left[u_{•,x}u_{•,y}u_{•,z}\right]}^{t}$ is the displacement vector on boundary •∈{L,R,T,B}, corresponding respectively to left, right, top, and bottom boundaries. $k_{x}$ (respectively $k_{y}$) is the wavenumber along *X* (respectively *Y*) axis. The dynamic equilibrium is then defined by the equation:

(3) $$\begin{array}{c} Du=F\\ \mathrm{where}\;D=-\omega^{2}M+\left(1+i\eta \right)K \end{array}$$

with M and K the respective matrices of mass, and stiffness, D the dynamic stiffness matrix, ω the system pulsation, η the damping coefficient, and u and F the respective vectors of nodal displacements and loads. The characteristic dimension of these matrices and vectors is the number of degrees of freedom (DOFs).

By applying the Guyan reduction—which differenciates boundary and internal elements —to Equation (2), the following eigenvalue problem is obtained:

(4) $$\left[S-\lambda_{i}I_{2n}\right]\Phi_{i}=0,\;\;\lambda_{i}=e^{j(k_{x,i}L_{x}+k_{y,i}L_{y})}$$

where S depends on D matrix coefficients with differentiated DOFs. For each eigenvalue $\lambda_{i}$, $\Phi_{i}=\left(\begin{array}{c} u_{L}\\ -F_{L} \end{array}\right)$, $I_{2n}$ is the identity matrix of size 2n, where *n* is the number of DOFs.

By solving Equation (3) for Floquet periodic solution (See Equation (2)), one can derive the dispersion diagram of the structure.

Practically, the direct WFEM is used here in order to get the dispersion curves. That is to say, the wavenumber $\lambda_{i}$ is fixed, and Equation (4) is solved to find the eigen pulsations $\omega_{i}$, and thus the eigenfrequencies $f_{i}$. The full dispersion diagram is obtained through sweeping $k_{x}$ and $k_{y}$ along the IBZ.

## 3. Results

### 3.1. Infinite Structure

In this section, an infinite plate in the XY plane is considered. Using the Floquet–Bloch theorem mentioned in Section 2.6, we know that a single periodic pattern is enough to model the whole structure’s dynamics, with greatly reduced computation cost. The same pattern will thus be used for statics but with simple periodic boundary conditions instead. Figure 2b and Figure 4b illustrate the concerned patterns. In the *Z* direction, plane strain approximation is assumed.

#### 3.1.1. Static Study

First, we will analyse the static compression of the infinite 2D structures, which gives the stress–strain curve of the structure. These results also define the different states of compression used as initial geometries for the dispersion calculations.

##### 3.1.1.1. Boundary Conditions

A uniaxial compression is considered along *Y* axis. To that end, a displacement is imposed on the top and bottom boundaries. Since we model a single pattern, periodic boundary conditions are added:

⊳ $u_{B,y}=-u_{T,y}=u_{0}/2$
⊳ $u_{R,y}=u_{L,y}$
⊳ $\frac{\partial u_{L,x}}{\partial x}=\frac{\partial u_{R,x}}{\partial x}=0$
⊳ $u_{T,x}=u_{B,x}$

where $u_{•}={\left[u_{•,x}u_{•,y}\right]}^{t}$ is the displacement vector along the boundary •∈{L,R,T,B}, respectively associated with left, right, top, and bottom boundaries. $u_{0}$ is the total compressive displacement imposed along *Y* axis. The effective strain is thus derived from $\varepsilon =\frac{u_{0}}{L_{Py}\left(\varepsilon =0\right)}$.

##### 3.1.1.2. Instability

In numerical simulations, symmetric boundary conditions applied to a symmetric model will always result in a symmetric solution. On the other hand, buckling corresponds to a loss of symmetry in the tangential stiffness matrix. This implies that in our case, the computation will give a purely axial compression, and buckling should not appear. In order to initiate buckling in the structure, an imperfection can be inserted to break the symmetry by a slight amount. To that end, a linear buckling analysis is first performed to obtain the first—and thus most likely—buckling mode. A small fraction (less than 1${\;}^{0}\;{/}_{00}$ of $L_{Px}\left(\varepsilon =0\right)$ in our case) of this mode’s displacement field is introduced in the initial structure.

##### 3.1.1.3. Results

**Figure 10:**
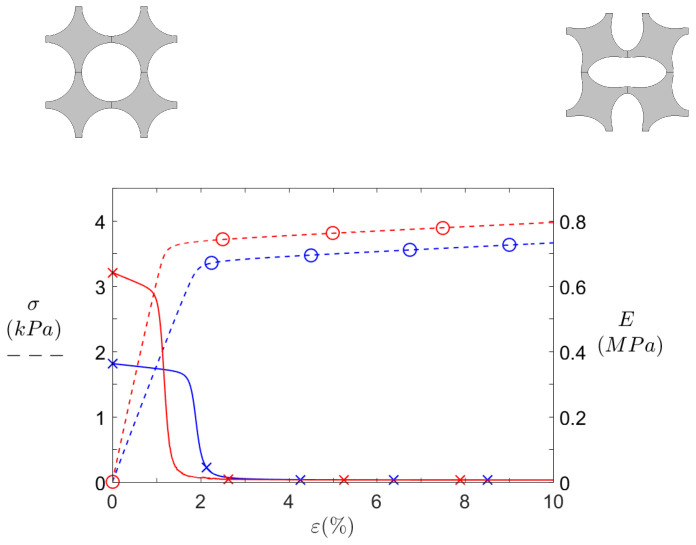
Static behaviour of $S_{0}$ (blue) and $S_{R}$ (red) patterns under axial compression. 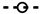 Effective stress σ and 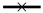 effective Young’s modulus *E* against compression axis (*Y*).

The first simulation to run is the static compression study, which gives the stress–strain curve of the structure. Results for the $S_{0}$ and $S_{R}$ configurations are shown in Figure 10.

At first, the effective stress curves (dashed lines) may look like trivial homogeneous yielding material traction curves, but the fact that they depict compression curves in the elastic domain makes them way more interesting. Two distinct trends can be observed on each curve: an initial slope relatively stiff compared to the second flatter one. To highlight this behaviour, the effective stiffness moduli $E=\frac{\Delta \sigma }{\Delta \varepsilon }$ (solid lines) are plotted against the compression axis. These curves feature two stable levels, the first one corresponding to a pre-buckling state and the second one associated to post-buckling stiffness. The latter is almost equal to zero, leading to a stress plateau. Though not shown here, this plateau continues until the cells collide with their neighbours, thus increasing the global stiffness again.

Now comparing the two designs, it can be seen that the initial stiffness is higher for $S_{R}$ than for $S_{0}$. This is less linked to the resonators themselves than to the ligaments shortening. In any case, a stiffer material usually entails a poorer isolation performance, so that any superior performance of $S_{R}$ compared to $S_{0}$ would come from the resonators inclusion.

Even though the $S_{R}$ design is stiffer, it buckles around 1% strain, before $S_{0}$ which does so at 2% strain. In short, the force required to buckle is higher for $S_{R}$, but its critical strain—at which it buckles—is lower. It is finally surprising to notice how both effective stiffness moduli get almost identical for strains above 3%.

#### 3.1.2. Dynamic Study

##### 3.1.2.1. Computation Parameters

The commercial finite elements software COMSOL Multiphysics^®^ is used in this study.

The software can generate meshes of various refinements automatically, but in order to reduce the computational cost, some parameters were defined manually. In particular, a nice mesh in the ligaments is paramount, as they are thin and sustain the greatest strains in the structure. A convergence study allowed one to determine a good balance between computational cost and accuracy. In the end, five elements were defined in the thickness at the thinnest point of the ligaments. The mesh was then generated automatically using an advancing front algorithm to tesselate triangular elements whose size varied between 1.12 μm and 0.3 mm, with a maximum growth rate of 1.2 and a curvature factor of 0.25.

One can note that the imposed displacement boundary condition implies boundaries, and thus a finite structure. Although it is incoherent with the infinite periodicity assumed in dispersion calculations, we suppose the compression to occur very far from the studied pattern, so that side effects can be disregarded.

It should be finally noted that infinitesimal strain theory being inconsistent here, geometric non-linearity is assumed in the computations.

##### 3.1.2.2. Dispersion Calculation

**Figure 11:**
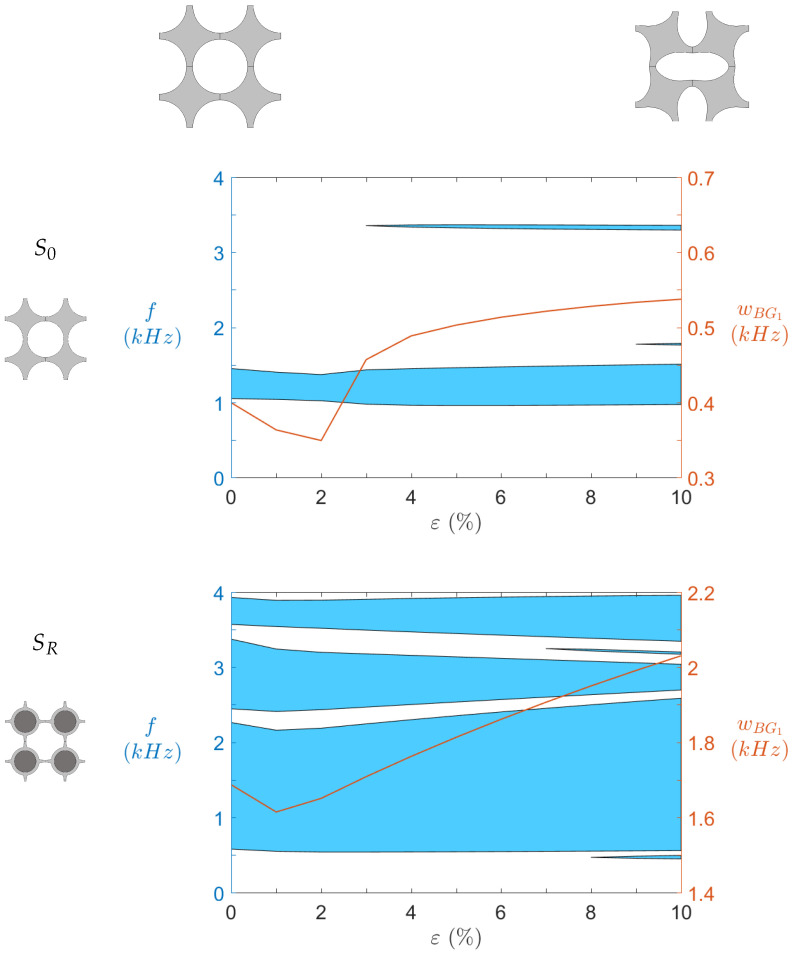
Evolution of omnidirectional bandgap ranges as function of imposed static strain for both geometries. 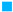 Bandgaps and 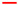 width $w_{{BG}_{1}}$ of the main bandgap.

By running computations on the five branches mentioned in Section 2.5, we achieve a good approximation of the bandgap structure of each design. In practice, we mainly focus on omnidirectional bandgaps under 2 kHz; some particularly large bandgaps led us to broaden the range up to 4 kHz. In addition, judging by the static curves, it is interesting to see how precompression affects the dispersion properties of the material. Such effect is displayed in Figure 11. The results are striking. The bandgaps exhibited by $S_{R}$ (bottom graph) are more numerous, wider, and at lower frequency than their $S_{0}$ (top graph) counterparts. Almost all frequencies between 500 Hz and 2.8 kHz are part of a bandgap. A narrow pass band exists around 2 kHz (at low strains) so the isolation is not perfect, but such a band contains very flat modes, that it to say modes with very low phase velocities $v_{\phi }=\frac{\omega }{k}$, making them easier to handle via conventional damping material.

Furthermore, the precompression level displays a significant effect on both geometries. This effect is highlighted by the red curves indicating the first low-frequency bandgap width $w_{BG_{1}}$. While its value for $S_{0}$ is strongly increased by the buckling (at ε=2%), it does not change much after 3% strain. A narrow second bandgap appears around 3.35 kHz after buckling, but does not get much larger with increasing strains. The third one appearing around 1770 is even thinner. On the other hand, $w_{BG_{1}}$ increases steadily after the buckling point of $S_{R}$, until reaching a width greater than 2 kHz for large precompression levels. An important feature of the $S_{R}$ design is the second wide bandgap, slightly above the first one. It is wideband—between 340 Hz and 930 Hz depending on the strain—and close to the first bandgap—the difference between them is around 170 Hz. It seems to be bound to the first bandgap, as its width decreases at almost the same rate as $w_{BG_{1}}$ increases. The same remarks can be made about the third large bandgap around 3.7 kHz (its wide varies between 360 Hz and 610 Hz. Narrow bandgaps appear for $S_{R}$ as well, in particular the one between the two first wide bandgaps. It makes the passbands even thinner, and could lead to filtering or wave steering applications.

As a result, the dispersion diagrams show that the locally resonant design seems to substantially improve the isolation performance of the metamaterial. However, these results are obtained for an infinite periodic plate. Therefore, to confront them to numerical and experimental tests of a finite structure is of interest.

### 3.2. Finite Structures

This section deals with the finite structures tests. The designs described in Section 2.2 are modelled to validate the results obtained in Section 3.2. Tridimensional models are computed with a *Z* depth of 40 mm, just like the experimental samples. Finally, experimental results are compared to computations.

#### 3.2.1. Static Study

Just like the infinite plate case, we first compute the stress–strain curve of the structure. Note that imperfections are still introduced in the symmetric model, as described in Section 3.1.1.2.

##### 3.2.1.1. Boundary Conditions

The finite size of the structure entails major changes in the boundary conditions:

⊳ Left and right boundaries are not constrained anymore, that is to say: $F_{R,x}=F_{L,x}=F_{R,y}=F_{L,y}=0$
⊳ As the top and bottom boundaries of the samples are clamped to rigid material, their *X* component is now null: $u_{T,x}=u_{B,x}=0$
⊳ Mimicking the experimental setup, the bottom boundary is also clamped along the *Y* direction: $u_{B,y}=0$, and the whole displacement is now imposed on the top boundary: $u_{T,y}=2u_{0}$

where $u_{0}$ is identical to the previous one (Section 3.1.1.1).

##### 3.2.1.2. Results

**Figure 12:**
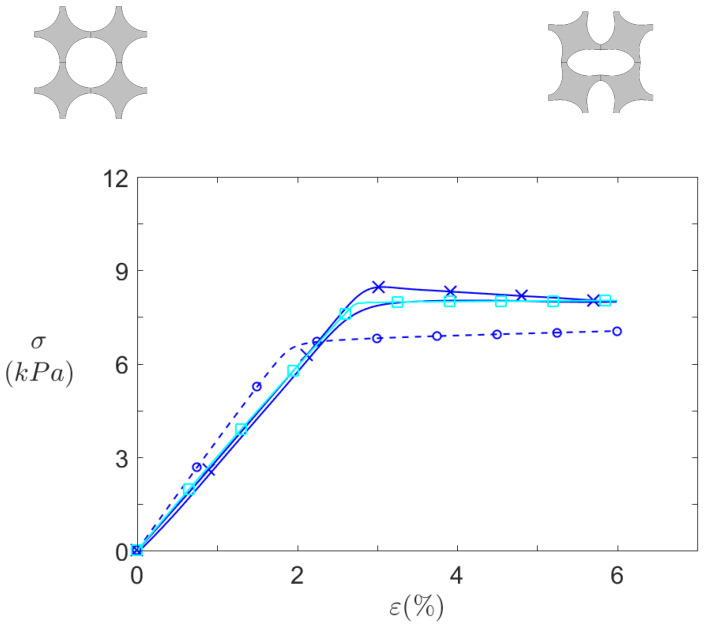
Static behaviour of $S_{0}$ pattern under axial compression. Effective stresses σ against compression axis (*Y*) for 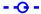 infinite plates computations, 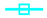 finite 8 × 7 structures computations, and 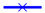 experimental 8 × 7 samples.

**Figure 13:**
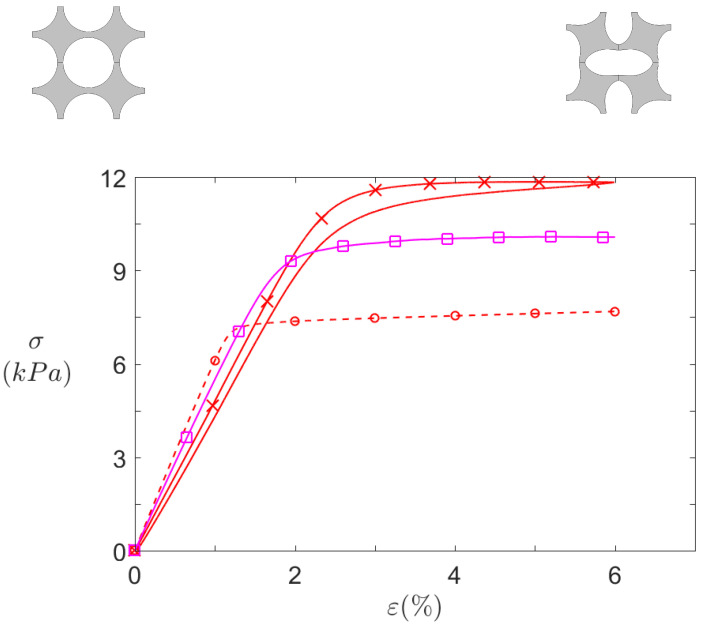
Static behaviour of $S_{R}$ pattern under axial compression. Effective stresses σ against compression axis (*Y*) for 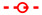 2D infinite plates computations, 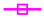 3D finite 8 × 7 structures computations, and 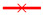 experimental 8 × 7 samples.

Figure 12 and Figure 13 summarise all the quasi-static compression results for $S_{0}$ and for $S_{R}$, respectively. Infinite plates computations, finite structures computations, and finite structures experimental measurements are respectively depicted by dashed lines with circles, dashed lines with squares, and solid lines with crosses.

By first comparing numerical data (circle-dashed lines and square-solid lines), it can be seen that transition from infinite to finite size significantly affects the structure behaviour. First the pre-buckling stiffness is slightly higher for infinite case, due to constraint difference along the *Z* axis. In 2D, the plane strain hypothesis prevents all deformation along *Z* axis, thus resulting in a stiffer material. On the contrary, the finite depth in the 3D computation allows some deformation, though small, along this axis.

**Figure 14:**
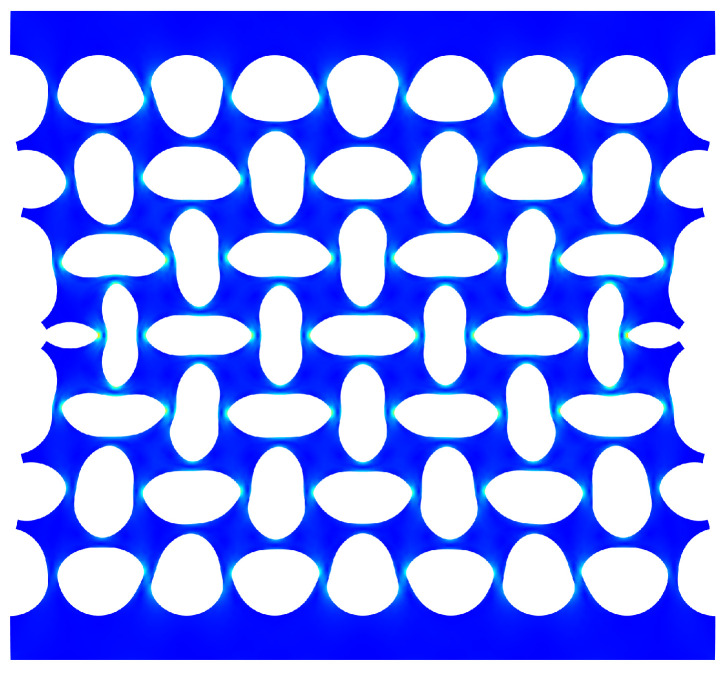
Computed deformed state of a finite 8 × 7 structure. The middle cells are much more deformed than the top and bottom ones.

Another difference between the both computed curves is the height of the zero stiffness plateau. In this case, it is caused by the difference in the top and bottom boundary conditions. By definition, the cells close to the clamped–clamped boundary conditions imposed on these boundaries (for the finite structures) are more constrained than in the periodic boundary condition imposed in the infinite plates. In finite case, these cells cannot deform freely and as a consequence, the deformation is not the same for all cells in the structure: the cells closer to the middle (regarding *Y* axis) of the structure are relatively less constrained, so that they are the first to buckle (See Figure 14). Overall, these additional constraints hinder the rotation of the top and bottom cells and thus increase the height of the zero stiffness plateau.

After buckling, the stiffness is virtually the same for both finite and infinite calculations, as they are very close to zero. The plateau of zero stiffness could be beneficial to the isolation performance, since a zero stiffness material could deform with no transmitted force. However, on the other hand, practical vibration sources excitation is usually an applied force. From an imposed force point of view, the plateau is invisible, and the structure strain state would simply "jump" from one side of it to the other, and zero stiffness would never be achieved. A logical solution would be to reach a strain state close to the plateau but slightly below it, so that the stiffness would have a finite value but be really small.

The experimental measurements shown in Figure 12 and Figure 13 differ from their numerical counterpart, but common features can be observed. First of all, the curves do not depict a simple compression (solid lines with crosses) but also the traction phase (solid lines without crosses) back to the initial point. For simulations, these two curves would be identical because the material model is purely elastic, but here a difference is observed. It is caused by the viscoelasticity of the silicone: during traction, it takes the material some time to revert to its initial state. However, this effect is very light in silicone, so that the linear material model is assumed satisfactory.

For $S_{0}$ (Figure 12), both the initial slope and the zero stiffness plateau are well captured by the 3D simulations, especially by the traction phase.

On the other hand, the results for $S_{R}$ (Figure 13) present much larger differences, as the initial stiffness is slightly lower than computations, but its plateau is higher. The reason for this gap might be the fabrication uncertainties associated with 3D printing. In this respect, dimensions of machined molds such as the $S_{0}$ mold are much more accurate than 3D printed ones.

Overall, the static results are satisfactory, as the dual stiffness behaviour—initial slope and plateau—is well captured.

#### 3.2.2. Dynamic Study

Lastly, we analyse the finite structures dynamic results.

##### Frequency Domain Analysis

**Figure 15:**
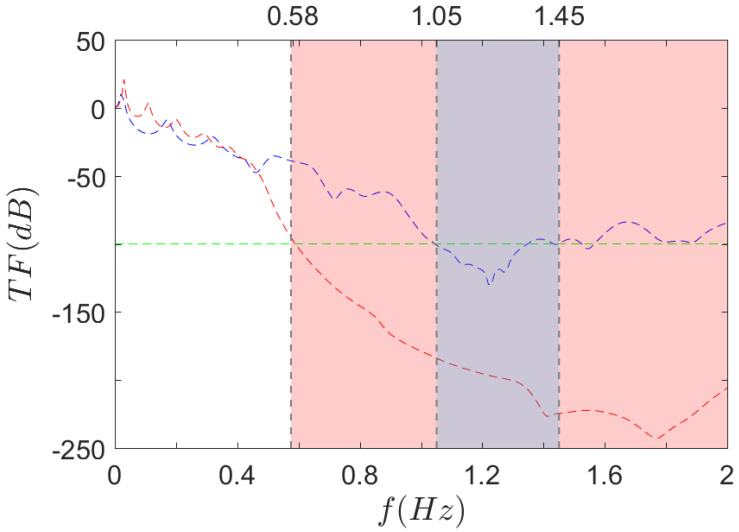
Computed FRF of 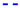 $S_{0}$ and 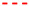 $S_{R}$ 3D structures. Omnidirectional bandgap ranges under 2 kHz for 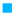 $S_{0}$ and 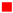 $S_{R}$, as found in the dispersion analysis.

To obtain the frequency response functions (FRF) of the finite structures, the computation parameters are the same as described in Section 3.1.2.1. The numerical boundary conditions are also identical to the static case (see Section 3.2.1.1), except for the top boundary, where a harmonic perturbation is now imposed. By sweeping the frequency range—from 0 Hz to 2 kHz in our case—the whole transfer function can be plotted. Figure 15 displays the transfer function $TF=F_{out}/F_{in}$ for both geometries (dashed lines) along with the omnidirectional bandgaps obtained in the dispersion calculation (Section 3.1.2.2) (coloured areas).

A first observation is that the $S_{R}$ curve is way below the $S_{0}$ on the major part of the frequency range. The mean TF value for $S_{R}$ is slightly higher in the low frequency modes’ range [0 Hz, 375 Hz], but beyond that point, the $S_{R}$ transfer function drops rapidly until a minimum (−242 dB) is reached for f=1.77 kHz. A similar drop is observed on the $S_{0}$ but mainly above 0.9 kHz and the minimum value is −130 dB obtained at f=1.23 kHz.

The other main result from Figure 15 is that the bandgaps seem to fit particularly low transfer function values. For instance, drawing a line at an arbitrary value of TF=−100 dB (green dashed line), it crosses the experimental curves at 590 Hz for $S_{R}$, and [1.05 kHz, 1.35 Hz] for $S_{0}$. Given that the corresponding bandgaps’ frequencies obtained by dispersion analysis are [0.58 kHz, 2.26 kHz] for $S_{R}$, and [1.05 kHz, 1.45 kHz] for $S_{0}$, the experimental data fit quite well—although slightly too high—for three values out of four. As for the upper limit of the $S_{R}$ bandgap, it is out of the scope.

To summarise the numerical results, a great correlation is found between the infinite plate and the finite structure dynamic computations, even if small differences remain. The final step is now to ascertain the actual performance of such materials through experimental measurements.

##### Experimental Validation

The measurements were realised with successive frequency sweep excitations. Even though it takes more time than a white noise excitation, it gives better coherence and allows one to easily tune the signal amplitude depending on the frequency. In particular, high amplitude at low frequencies can result in huge displacements at resonances. Therefore, a width of 100 Hz is chosen for each sweep, and all steps put together allow one to plot all the transfer functions, along with the phases and coherences.

**Figure 16:**
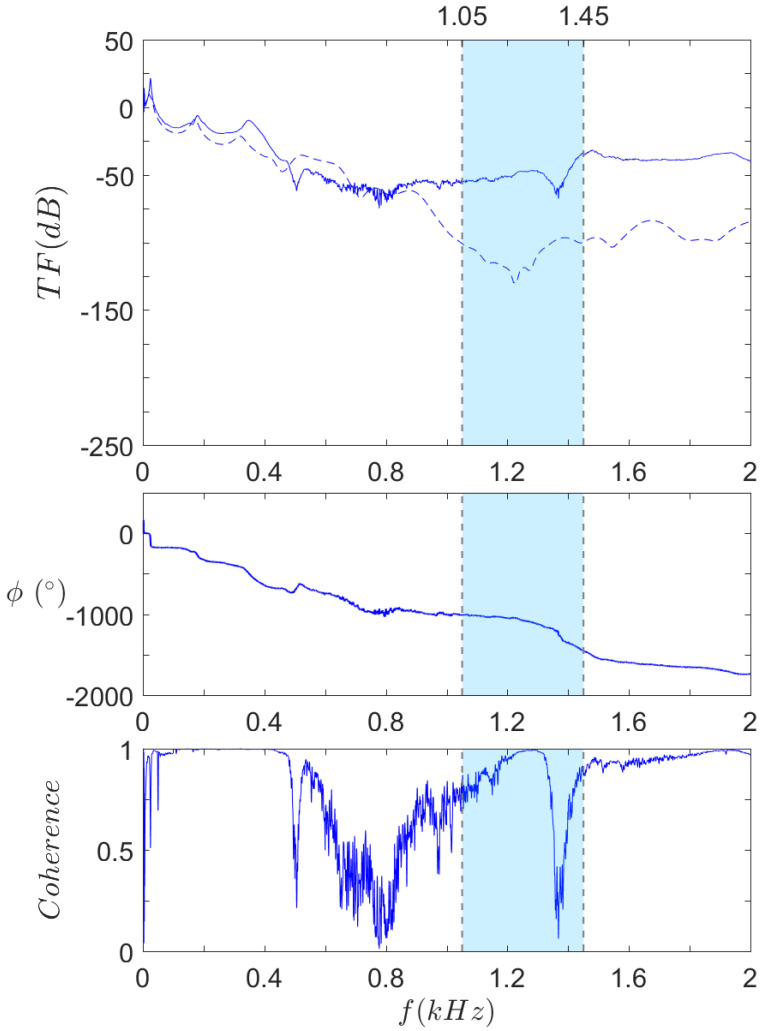
Transfer function (**top**), phase (**middle**), and coherence (**bottom**) of $S_{0}$. 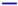 Experimental and 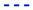 computed results. 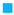 Omnidirectional bandgaps under 2 kHz, as found in the dispersion analysis.

**Figure 17:**
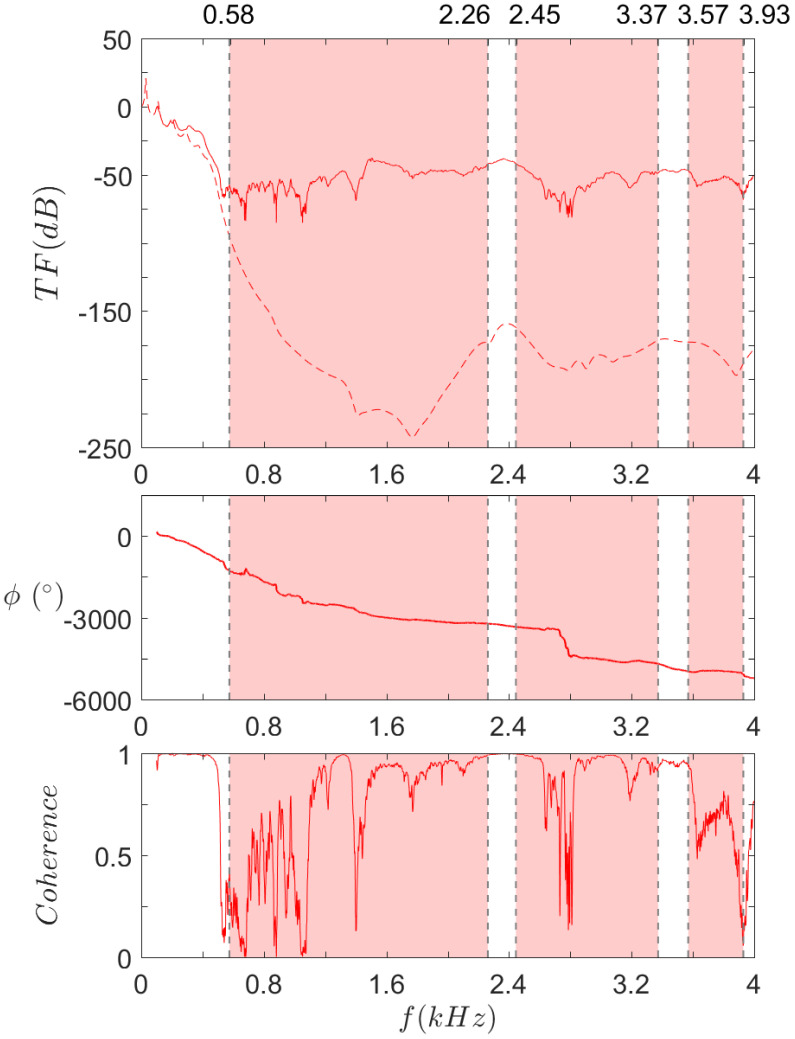
Transfer function (**top**), phase (**middle**), and coherence (**bottom**) of $S_{R}$. 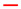 Experimental and 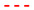 computed results. 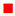 Omnidirectional bandgaps under 4 kHz, as found in the dispersion analysis.

**Figure 18:**
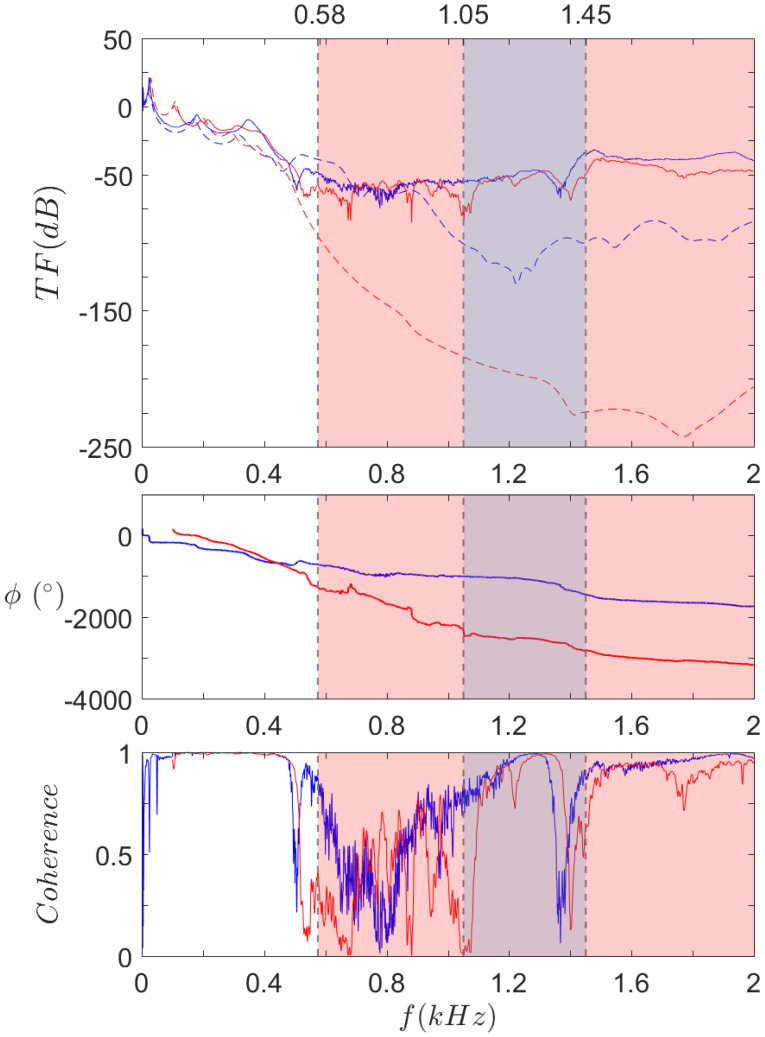
Experimental transfer functions (top), phases (middle), and coherences (bottom) of 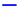 $S_{0}$ and 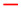 $S_{R}$, compared to their computed counterpart 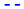 $S_{0}$ and 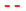 $S_{R}$. Omnidirectional bandgap ranges under 2 kHz for 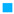 $S_{0}$ and 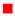 $S_{R}$, as found in the dispersion analysis.

The results for $S_{0}$, $S_{R}$, and both, are respectively displayed in Figure 16, Figure 17 and Figure 18. The frequency range is kept to [0,2] kHz, except for $S_{R}$. The previous results indeed showed that the upper limit of the first bandgap is above 2 kHz, so the range is extended to [0,4] kHz in this case.

Figure 16 shows that there is a good correlation of the first modes between experimental and numerical results, even though a small offset is noticeable, especially for higher frequency anti-resonances. For instance, the computation shows anti-resonances at 460 Hz and 1230 Hz, while the same anti-resonances occur at 500 Hz and 1370 Hz. This phenomenon could be caused by a difference between the material elasticity modulus in the numerical model and the real one, since the modes’ frequencies are directly proportional to the root square of the elasticity modulus. The levels of transfer function are quite similar until 900 Hz, but a great gap arises after this frequency: while the computation displays a sudden drop, shortly before the bandgap predicted by the dispersion analysis, the experimental curve keeps a steady level at that point.

Another remarkable aspect is highlighted by the coherence curve: the coherence level drops abruptly around certain frequencies. Experimentally, low coherence generally corresponds to a very weak response, such that the transmitted vibrations—that are coherent with the harmonic excitation—are hard to distinguish amid the measurement’s background noise. In Figure 16, the first and third drops perfectly match the anti-resonances mentioned above. As for the second one, its range is much wider, extending from 550 Hz to 1200 Hz. An anti-resonance is also predicted by the computations at around 720 Hz but that alone does not explain the considerable width of the phenomenon. A possible explanation is that this low coherence zone actually corresponds to the predicted bandgap but down-shifted. As to the reason why this shift occurs, it has yet to be fully understood. Future work will focus on answering this problem.

Figure 17 describes the same results for $S_{R}$. Like $S_{0}$, experimental low frequency modes seem to fit quite well the numerical ones. A transfer function drop is observed on both curves at 500 Hz, but while the numerical curve drops rapidly down to −200 dB, the experimental results stay around −60 dB. Instead, the coherence suddenly drops at that point. All indications are that the background noise is around −60 dB, so that any result theoretically below would be lost in the noise. Focusing on the coherence, one can see that some sudden changes match quite well bandgaps’ limits, in particular the lower limit of the first bandgap (0.58 kHz) and both limits of the third one (3.57 kHz and 3.93 kHz). However, other bandgaps’ limits do not follow this trend (for instance at 2.26 kHz and 2.45 kHz), and reciprocally, some frequencies theoretically included in a bandgap do not correspond to a low coherence level (see for instance at 1.3 kHz and 3 kHz). These incoherences could be linked to boundary conditions’ effects or material considerations such as rubber saturation and will be investigated in future work.

Finally, Figure 18 allows for the comparison of both $S_{0}$ and $S_{R}$ experimental results. First of all, one can notice much less difference than expected between $S_{0}$ and $S_{R}$ curves. Except for some features like low frequency modes and a few anti-resonances in the $S_{0}$ curve, both are quite similar.

The real difference between them is highlighted by the coherence curves. When looking at $S_{0}$ results, one can see that the coherence increases above 0.9 at 540 Hz, after the first drop at 500 Hz. On the other hand, $S_{R}$ coherence sticks to very low levels in that range and stays broadly below 0.5 until 1100 Hz, which represents a 600 Hz wide isolation band. Comparatively, the largest continuous isolation zone with coherence below 0.5 extends from 600 Hz to 900 Hz, that is to say a 300 Hz wide band. Therefore, the isolation gain obtained with $S_{R}$ geometry can be considered efficient on a large frequency band, compared to reference geometry.

Even though further measurements should be performed to confirm these results, what can be observed from these curves is that the experimental $S_{0}$ bandgap is wider than expected whereas the $S_{R}$ one is narrower, to the point that the improvement from the local resonators is not as great as expected. Nevertheless, the $S_{R}$ bandgap is twice as large as its counterpart, extending in particular near the low frequencies, thus achieving better isolation performance in this frequency range.

## 4. Discussion

To summarise, this article studies the isolation performance of two metamaterials, a classic auxetic one $S_{0}$ and a enhanced one $S_{R}$ with resonant inclusions. For these two geometries, static and dynamic computations have been carried out for both infinite plate assumption and 8 × 7 cells finite geometry, along with static and dynamic measurements on molded samples. The static study has shown that edge effects due to finite size entail substantial modification of the effective stress–strain curve for axial compression. A plateau of zero stiffness appears after buckling. The initial slope is also the same for both cases as well as for the experimental test, to a certain extent. The material model is not sufficient to capture the measured plateau levels, at least in the $S_{R}$ case. In any case, the static curves exhibit a deep stiffness drop triggered by buckling. This feature could be used to mechanically tune the structure properties and possibly greatly improve the isolation properties. The dispersion analysis has confirmed that the bandgaps are strongly affected by the precompression state of the metamaterial. In particular, their overall width and number tend to increase with strain. Compared to $S_{0}$, the resonant design exhibits huge improvement of the bandgaps, whether it be their width, number, or minimum frequency. Such bandgap widths have seldom been reported in literature.

Finite structures’ calculations confirm that such behaviour can also be observed in finite sized structures. Great transmission loss is seen at the bandgaps’ frequency ranges, and the $S_{R}$ design exhibits a much deeper transmission loss that $S_{0}$. Finally, vibration isolation tests have been performed on experimental samples. Although the difference between both samples is not as great as expected, the $S_{R}$ design does extend the bandgap near the low frequencies. Substantial isolation is achieved with a 600 Hz wide continuous bandgap reaching frequencies as low as 500 Hz. This study highlights the great isolation performance of auxetic metamaterials harnessing buckling and resonance effects. This could lead to better isolation systems, with advantages such as compacity, low frequency efficiency, broadband application, lightweight, and passivity (no external energy required). Vibrating machine isolation in industry, passengers’ and drivers’ comfort in transportation, or seismic protection of buildings are a few applications which could benefit from these types of metamaterials.

As prospects, the bandgap analysis could first be validated by a finer sweep in the IBZ and then broadened with unidirectional bandgap analysis and extended calculations for three-dimensional propagation. The stress levels inside the structure could also be compared to failure stress, in particular fatigue failure stress. Further work will also aim at solving the bandgaps shift observed in the dynamic study.

## Figures and Tables

**Table 1:** Materials’ properties.

**Material**	**Silicone**	**Steel**
E	0.97 MPa	200 GPa
ν	0.499	0.3
ρ	1150 kg/m^3^	7850 kg/m^3^

## Data Availability

The data are not publicly available due to confidentiality agreement with funder.

## Abbreviations

The following abbreviations are used in this manuscript:

BZ	Brillouin Zone
DMA	Dynamic Mechanical Analysis
IBZ	Irreducible Brillouin Zone
TF	Transfer Function
WFEM	Wave Finite Element Method
